# A Systematic Review on the Impacts of Climate Change on Coffee Agrosystems

**DOI:** 10.3390/plants12010102

**Published:** 2022-12-25

**Authors:** Christine Bilen, Daniel El Chami, Valentina Mereu, Antonio Trabucco, Serena Marras, Donatella Spano

**Affiliations:** 1 Department of Soil, Plant and Food Sciences, University of Bari ‘Aldo Moro’, 70126 Bari, BA, Italy; 2 TIMAC AGRO Italia S.p.A., 26010 Ripalta Arpina, CR, Italy; 3 Impacts on Agriculture, Forestry and Ecosystem Services (IAFES) Division, Euro-Mediterranean Center on Climate Changes (CMCC), 07100 Sassari, SS, Italy; 4 Department of Agricultural Sciences, University of Sassari, 07100 Sassari, SS, Italy

**Keywords:** coffee systems, production, climate change (CC), impacts, ecosystem services

## Abstract

Coffee production is fragile, and the Intergovernmental Panel on Climate Change (IPCC) reports indicate that climate change (CC) will reduce worldwide yields on average and decrease coffee-suitable land by 2050. This article adopted the systematic review approach to provide an update of the literature available on the impacts of climate change on coffee production and other ecosystem services following the framework proposed by the Millenium Ecosystem Assessment. The review identified 148 records from literature considering the effects of climate change and climate variability on coffee production, covering countries mostly from three continents (America, Africa, and Asia). The current literature evaluates and analyses various climate change impacts on single services using qualitative and quantitative methodologies. Impacts have been classified and described according to different impact groups. However, available research products lacked important analytical functions on the precise relationships between the potential risks of CC on coffee farming systems and associated ecosystem services. Consequently, the manuscript recommends further work on ecosystem services and their interrelation to assess the impacts of climate change on coffee following the ecosystem services framework.

## 1. Introduction

Coffee is the world’s second-most consumed beverage. There is no exact established date for the first time humankind consumed coffee, but different anecdotes and legends date back to the ninth century [1]. It could have been that Arabs discovered it in Africa and introduced it in the trade across the Silk Road, which linked Africa to the Arabian Peninsula through the Red Sea [2].

The global coffee trade depends on two species: Arabica (*Coffea arabica*), which makes up about 60% of traded coffee, and robusta (*Coffea canephora*), which makes up the remaining 40% [3], even though the taxonomy of the genus *Coffea* presents 130 species and seven intraspecific taxa [4,5,6].

**Figure 1:**
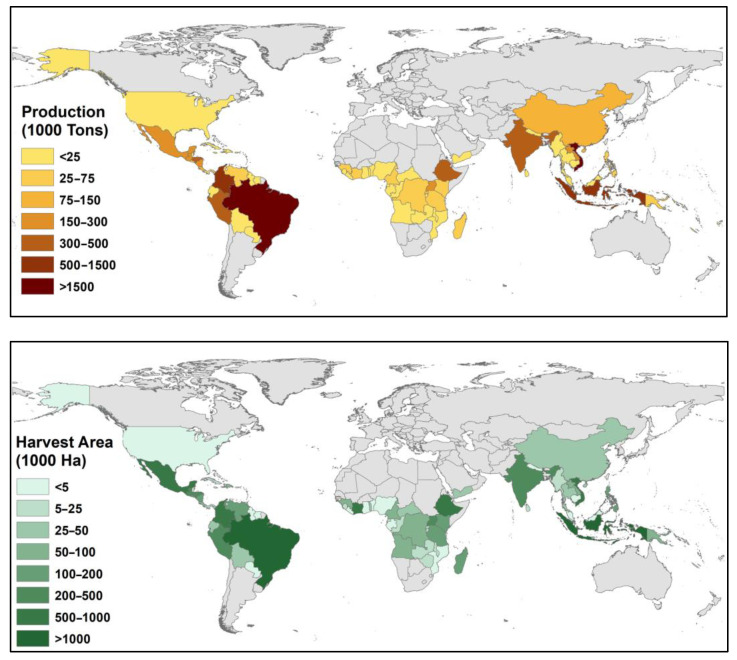
World coffee production (**top**) and harvested area (**bottom**) in 2019 [7].

According to world statistics, the average world production of coffee exceeded 10 million tonnes, and the total harvested area is over 11 million hectares [7]. The Americas produce over 55.5% of the whole world’s production, followed by Asia with 31.9% [7], with the top coffee-producing countries being Brazil, Vietnam, Indonesia, Colombia, etc. (Table 1); however, more than seventy different countries are valuable producers (Figure 1).

Despite being a strategic commodity with a historic and robust supply chain, coffee production is a fragile sector tormented by enormous challenges. Climate change (CC) is the most pressing issue, expected to reduce worldwide yield and decrease coffee-suitable land by 2050 [8,9,10], and requires timely and effective agronomic changes to reduce the potential risks and ensure the long-term viability of coffee production [11]. Similar to all other agrosystems, the consequences of climate change on coffee production triggers other risks associated with the soil, water, crop, and nutrient management: drought, salinity, biodiversity decline, suitability losses, change of species seed availability, resistance to abiotic and biotic stressors, etc. [11,12]. In fact, sustainable coffee systems may provide several ecological services, such as maintaining soil fertility, biodiversity, and carbon sequestration, and regulating pests and diseases [8,12]. Environmental challenges such as the deterioration of soil health, biodiversity conservation (shade trees, flora, and fauna), and pollution are serious hazards to ecosystem functioning [12,13].

Moreover, the vulnerability of coffee growing areas is influenced by several factors such as, among others, land size and income level, labour availability, postharvest infrastructure, access to market, negotiation capacity, technical and financial assistance, etc. (IPCC, 2022). Some of the topics discussed in the literature comprise worsening socio-economic conditions of farmers, increase in poverty levels, a decline in well-being [14,15], and impacts on infrastructure and logistics [16,17]. To overcome these obstacles, coffee growers must implement integrated management practices on their farms to ensure the long-term viability of coffee production [12]. As highlighted in the AR6 IPCC report (IPCC, 2022), the interactions between climate, ecosystems, and human society underlie the expected risks and must be considered as a whole and in all their complexity to meet the climate-resilient development goals, including adaptation, mitigation, and sustainable development goals.

According to the literature, climate change impacts influence different ecosystem services [18,19] and threatens the sustainability of coffee agrosystems, especially “Arabica” coffee, which is more susceptible to climate variability [20,21,22]. On the other hand, impact category and intensity determine adaptation practices and resilience strategies to cope with expected variability and changes.

In this context, the challenge is to identify and implement actions to cope with these impacts and promote a resilient and sustainable development (in terms of productivity and quality) of the entire coffee production chain. Stakeholders and decision-makers need consolidated scientific evidence to design and implement successful adaptation practices and resilience strategies for coffee production in environmentally sensitive regions.

A systematic review is an evidence-based, robust, rigorous approach reproducible in all fields. It is used to draw scientific conclusions reducing susceptibility to bias [23]. Recently, it has had several applications in agrosystems and climate change science [24,25]. Previous reviews have assessed the impacts and adaptation of climate variability and change on coffee production [21,26,27,28] and have indicated increasing interest in its climate-related risks. However, the authors failed to present the results according to the impact types using the ecosystem services framework proposed by the Millennium Ecosystem Assessment [29]. This framework may prove fundamental in decision-making to highlight the sustainability of different adaptation and resilience practices and strategies to sustain coffee crop production. In view of the global climate resilience and sustainable development goals [8], it is crucial to deepen these aspects for all agricultural systems, and particularly for crops such as coffee that have significant social, economic, and environmental impacts, to better support the decision making in the short- to medium-term period.

Therefore, this research adopted the systematic review approach to update the literature available on the impacts of climate change on coffee production, assessing the results following the ecosystem services framework applied in [30] used to review the impacts of climate change on sugarcane production.

For this purpose, the authors formulated the primary research question of this systematic review following a compromise between the holistic (widening the number of records) and the reductionist approach (limiting the number of records) [31]. The primary research question is:

“What are the impacts of climate change on ecosystem services of coffee production?”

## 2. Results

**Figure 2:**
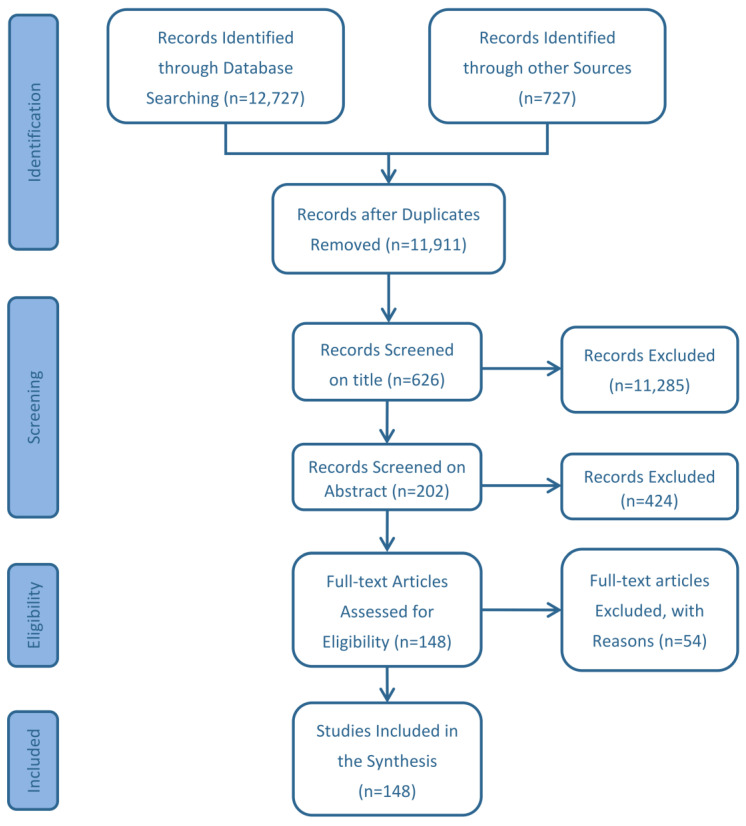
Flow diagram of the systematic review process (after [32]).

The systematic review results are divided into different stages, as summarised in (Figure 2). We based the initial filtering on the title of the literature source and the second filter on the content in the abstract. We undertook the full-text review only for articles that passed all criteria.

### 2.1. Bibliometric Analysis

At the end of the screening process, a total of 148 references were considered eligible for full-text evaluation and were included in the data synthesis, which was organised by regions/countries, coffee species, and types of investigational methods. These articles contained relevant information about the direct or indirect effects of climate change or climate variability on coffee production that could be used and evaluated in the review.

We assessed the selected references from the literature using a network analysis tool (InfraNodus) to check their relevance and to depict relations among documents’ titles and their centrality. Network analysis, though not directly cognitive, offers considerable insight into how shared cognitive structures and content spread and the conditions that affect such spread. Network analysis is helpful in many living application tasks. It helps to depict and graphically visualise the structure of a relationship in social networks, a process of change in natural phenomena, or even the analysis of biological systems of organisms [33,34].

**Figure 3:**
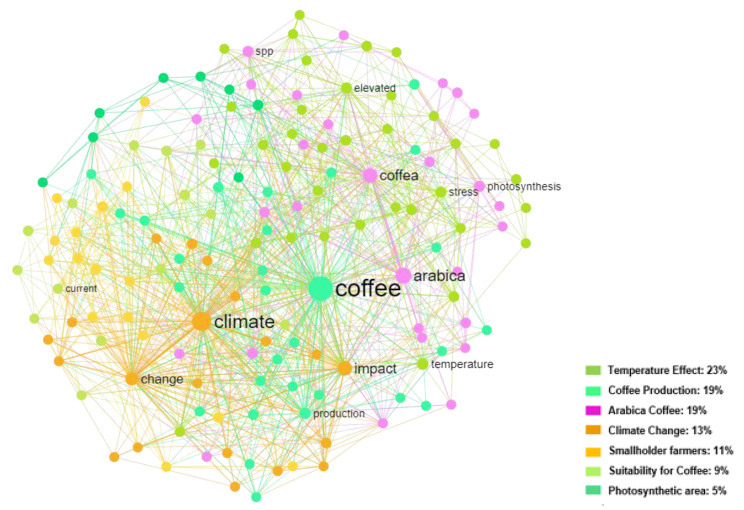
Concept network mapping for references’ titles screened for the final review and the corresponding keywords. The main topical clusters are shown on the map legend, each of which is distinguished by a particular colour. Thus, the three factors that have the largest influence—represented by the larger circles—are coffee, climate, and Arabica.

We used a web-based analytical engine to develop the network (https://infranodus.com/), which stems the words to reduce the redundancy and complexity, using the Krovetz Stemmer algorithm, and subsequently converts the text into a network after a two-pass analysis [35,36,37]. The graph shows a high degree of centrality, the level of eigenvector, betweenness and closeness of this centrality between the following keywords generated (Figure 3), which confirms the consistency and accurateness of the selection process.

**Figure 4:**
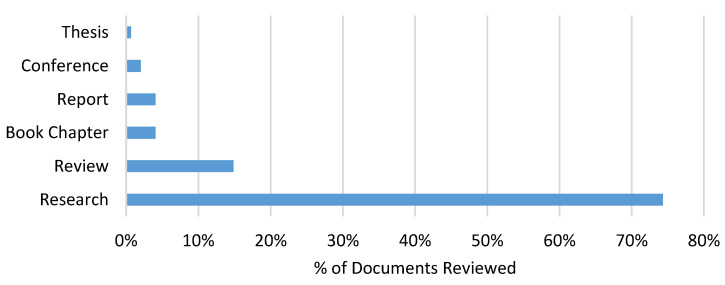
Research papers (74.3%), reviews (14.9%), book chapters (4.1%), conferences and reports (4.1%), and theses (0.7%) are the types of manuscripts included in the review.

The collected data show an increase in the number of recently published articles (for example, in 1980, there was only 1 article in the literature, however, in 2019 and 2020, the number increased to 14 and 25 articles, respectively), thus reflecting the increased interest in the potential effects of climate variability and change on coffee production. Most of the included documents are original research papers (74.3%), with a few reviews, book chapters, conferences, reports, and theses (Figure 4).

### 2.2. Coffee Species

**Figure 5:**
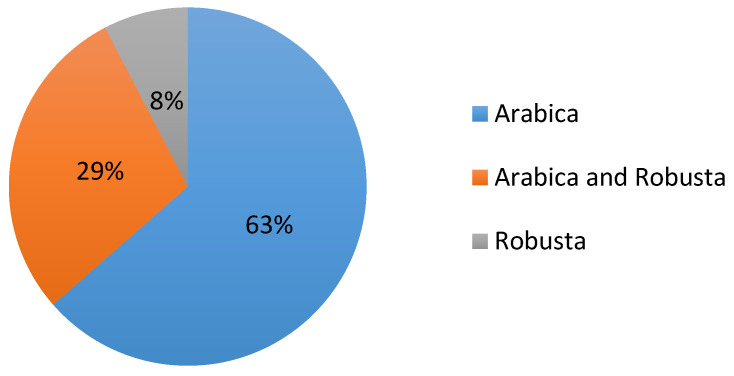
The coffee species reviewed in the research: 75 records, which account for 63%, considered the impact of climate change on the *Coffea arabica* species. In contrast, only nine documents (8%) of the research explored the effect of CC on *Coffea canephora* (robusta species). Thirty-four manuscripts (29%) included both species in their research.

According to the review of related literature, Arabica was the most-studied coffee species (75 documents), followed by publications on both Arabica and robusta coffee species together (34 documents). Only a few focused on robusta species (nine documents), even if it accounts for approximately 40% of global production [38] (Figure 5). Moreover, the coffee species was undefined in the 30 other documents.

These results may be attributable to the fact that most of the reviewed manuscripts were conducted on the American continent, where Arabica has the most extensive diffusion. Moreover, even though *Coffea arabica* (Arabica) and *Coffea canephora* (robusta) contribute the most to worldwide coffee production, Arabica coffee is grown in more than 80% of coffee-growing nations and has a relatively higher demand for materials with enhanced beverage quality [39,40]. Another aspect is that Arabica coffee is more sensitive to climate factors than robusta coffee and will be more affected by climate change [41].

Robusta coffee may be marginally more resistant to temperature increases, although it is more cold-sensitive [28]. Moreover, robusta may be susceptible to increasing intraseasonal temperature variability [41], which poses other negative impacts linked to climate change. While bioclimatic suitability for robusta production is projected to decline altogether by some global studies, there is a general lack of large-scale research on the climate-sensitive flowering and growth phases of robusta. Future research is required to determine its optimal temperature ranges more precisely to enhance yields [27,42].

### 2.3. Geographic Distribution

America, Africa, and Asia are the three continents whose major regions are outlined in the review. The majority of articles (90) were from the Americas, followed by Africa (39 records) and Asia (19 papers). In addition, four of these evaluated articles covered the American and African continents. In comparison, seven articles involved the three continents.

Specifically, South America predominated, with most research conducted in Brazil (51 records), followed by Colombia (12 documents). During the 2016 harvest, Brazil produced approximately 50,3 million sixty-kilogram bags of coffee, of which 42.5 million contained Arabica coffee and 7.8 million contained conilon (robusta) coffee [43]. North and Central America followed with 15 and 12 records, including Mexico and Nicaragua. Moreover, most African manuscripts reviewed were conducted in Ethiopia (17 articles), Kenya, and Tanzania (10 records each). Ethiopia is the largest coffee producer in Africa and the world’s third-largest producer of Arabica coffee by volume and value after Brazil and Colombia [44]. Research from Asian nations was limited, with Vietnam and Indonesia dominating (eight documents in each country), even though this continent is the second-largest producer of coffee (31.9% of the whole world’s production). The remaining research was limited to one or two papers per country on each of the three continents.

The predominance of research in the Americas may reflect that more than half of the world’s coffee is produced by the top ten coffee-producing countries [27]. However, more research is required to support the sustainable development of coffee in regions with high production levels, especially in communities that rely heavily on coffee cultivation. Similarly, most reviewed records concentrate on national and subnational production scales. According to reports, available data at large spatial scales are inadequate and uncertain [27].

### 2.4. Methods to Estimate Climate Change Impact

The review showed various research methods used by authors to analyse CC impacts on coffee production. Research employing quantitative methods predominated over qualitative methods (≥60 manuscripts vs. 13 records), with seven articles applying mixed methods.

Qualitative methods such as interviews, focus groups, surveys of households, and document analysis were used to identify how climate change or climate variability affects coffee production directly or indirectly through the spread of pests and diseases. This methodology can provide context-specific data, such as the perceptions and experiences of local farmers and their responses to climate change. This is an important knowledge base in order to identify coffee production system adaptation [27].

Quantitative methods comprised a variety of modelling approaches designed to investigate the impact of climate variability and change on coffee production systems. Numerous manuscripts employed machine-learning techniques, notably Maximum Entropy (MaxEnt), with the majority focusing on the current and future suitability driven by climate for coffee cultivation [45]. MaxEnt is a niche modelling method involving species distribution information based solely on known presences. It is a general-purpose technique for making predictions or inferences of occurrence probability or fitness to the environment from incomplete data.

Bioclimatic models were among the other types of ecological niche modelling used. Fewer manuscripts utilised statistical analysis and econometric models to investigate the direct effects of climate change or climate variability on coffee production or the distribution of pests and diseases affecting coffee farming. Several references employed alternative modelling techniques, such as agricultural zoning and other species distribution simulation models. In the current research, a limited number of manuscripts (three to four) used mechanistic or process-based models to analyse potential climate-driven effects on coffee production.

The bioclimatic modelling approach determines the likelihood of occurrence or non-occurrence in non-sampled areas by relating known occurrences and absences with environmental variables [22]. An econometric model integrates climate and economic variables and is validated using statistical analysis before being used to project coffee production under various climatic circumstances [46]. On the other hand, agricultural zoning is developed based on integrating crop growth models, climate and soil datasets, decision analysis methodologies, and geoprocessing tools [47].

While studies using MaxEnt or other bioclimatic modelling approaches have estimated the potential distribution in areas suitable for coffee production under current and future climates, they have yet to incorporate phenotypic plasticity [48] or mechanistic processes to predict more dynamically the responses [49] of the coffee plant to climate change or the effect of adaptation measures. Such models could be a valuable tool for gaining a deeper understanding of climate change effects, including the impact of altered microclimates and change in resources caused by management practices on coffee production systems, by enabling the analysis of interactions between climate, soil, and coffee plant parameters [49].

Current references on the effects of climate change on the suitability of coffee-growing regions use several climate models with varying levels of spatial resolution, ranging from 30 arcsec (1 km^2^) to 30 arcmin (50–60 km^2^), which may account for the wide variation in reported estimates. Coarse spatial resolutions may not capture local features, such as the heterogeneous topography of coffee-growing regions. To a certain extent, downscaling and interpolating coarse climate projection data may limit uncertainties and errors, which are more prominent in agricultural landscapes with topographic heterogeneity [50,51]. Climate models with low temporal and spatial resolution make it difficult to link climate scenarios to biological responses, such as pest or disease development, which requires daily or even hourly data [52,53]. Using models with high spatial and temporal resolution would improve climate impact simulations by facilitating the capture of non-homogenous topographies and thus more accurately represent microclimatic characteristics [20] and reduce uncertainties through the use of more refined climate data [53].

According to current research, the assessment of uncertainties associated with climate variables and scenarios, interpolation processes used for climate projection data, model parameters, socioeconomic factors, and interactions between the coffee plant and its environment are still underdeveloped. A few references [49,54,55] analysed uncertainty partially or explicitly. Incorporating outputs from a multi-model ensemble to provide improved forecasts has been suggested to reduce uncertainty caused by erroneous representations of suitable climates [41,44,56]. It should be noted, however, that ensemble modelling may produce inaccurate results due to errors and biases in individual species distribution models [57].

### 2.5. Potential Impacts of Climate Change

This review also assessed different groups of impact sources, comprising climate change and variability, extreme temperature (low or high temperature), variability in precipitation patterns (drought and waterlogging), and elevated atmospheric concentrations of carbon dioxide ([CO_2_]). The physiological responses, disease infestation, bioclimatic suitability of yield, and producer welfare are the four major subgroups of climate change impacts that have been assessed. Alternately, these impacts could be classified as “direct” when they affect coffee yield and production and “indirect” when they affect the severity of diseases, the nutritional quality of coffee, and the stakeholders.

Numerous manuscripts have examined the impacts of climate change and variability on coffee production. In addition to temperature and precipitation variations, indicating a growing awareness of their potential consequences, most references have reported adverse effects. Some of which also reported mixed results. However, multiple citations have focused on the impact of elevated [CO_2_] levels. Most reports indicated the positive fertilisation effect following enhanced [CO_2_] to partially offset the adverse effects of growing temperatures and droughts.

#### 2.5.1. Impact of CC on Coffee Yield and Production

Of all the references investigating the direct effect of climate change on coffee yield or production (42 papers), 35 indicated negative impacts. Four studies reported mixed results, and three studies revealed positive effects. The analyses identified an overall reduction in coffee yield in the three continents (America, Africa, and Asia). Most yield decreases and losses were reported primarily in the Americas, where the global loss could be as high as 70% [58]. Intriguingly, one manuscript done in the provinces of Indonesia and Vietnam (Southeast Asia) quantified robusta’s optimal temperature range for production and showed it might present losses against climate change. The data indicated a decline in the production potential of *Coffea canephora*, placing a multibillion-dollar coffee industry and the livelihoods of millions of farmers at risk [42].

However, references with contradictory findings have included a positive offset by the [CO_2_] fertilisation effect, resulting in a slight net increase in the average Brazilian Arabica coffee yield. On the other hand, decreased coffee production in Mexico (central Veracruz) led to a rise in its economic value but a decline in socioeconomic indicators [55,59].

#### 2.5.2. Impact of CC on Land Suitability

Regarding the land suitability for coffee production, the research (a total of 54 papers) revealed an overall change in the suitability of the current coffee-growing areas. The change will mainly affect suitability negatively, where a general pattern of decreasing suitable areas and suitability within these areas will occur (33 papers). Large portions of important coffee-producing nations, including Brazil, Vietnam, Honduras, and India, will become unsuitable. The most significant reductions in suitability are expected in Ethiopia, Sudan, and Kenya (up to 90% reduction by 2080 [60]), Puerto Rico (84% by 2070 [51]), Mexico (98% by the 2050 s [61]), and Latin America (88% by 2050 [62]). In contrast, future changes in the suitability zones will alter the geographical distribution of potential optimal sites, increasing the suitability and productivity of vast regions formerly unsuitable for coffee. Several studies predict expanding coffee-growing areas in South America, East and Central Africa, and Asia [41,63,64,65]. Some regions projected to be favourable for coffee cultivation are open land, such as those in East Africa [41,64], while others, especially in the Amazon basin, Asia, and Central Africa, are currently under forest cover [41], protected areas [65], or other agricultural land uses [63].

Many studies predict that suitability will shift to higher altitudes where temperatures are cooler, but these zones will likely adversely affect the ecosystem. The risk of converting high-altitude forests and protected areas into agricultural land will increase as coffee-growing areas migrate higher [45]. Changes in the suitability of coffee species accompany the relocation of producing regions. Other regions will become less climatically suitable for growing Arabica coffee (in South and Central America, Africa, and Asia) but more suitable for growing robusta coffee [66]. At least 83% of the total future coffee-growing area meets the requirements for robusta cultivation, but only 17% (±6%) meets requirements for Arabica [63].

Furthermore, Mesoamerica would experience a decrease of up to 30% in the area suitable for Arabica coffee, with the most considerable losses would occurring in Mexico (29%) and the smallest in Guatemala (19%). The Andean nations would lose 16–20% of their current Arabica coffee-suitable land, while Brazil would lose 25%. Indonesia’s area suitable for producing Arabica coffee would likely decrease by 21–37% [64]. In Ethiopia and South Sudan, the population of wild Arabica coffee will be reduced by at least 50% by 2088 [67]. Destruction of natural coffee habitats consequently impacts coffee genetic resources and livelihoods [68]. The demand for robusta coffee could be met without incurring forest encroachment in most regions. Future cultivation of Arabica can be accommodated, but only at the expense of natural forest loss, which has negative repercussions for carbon storage and is likely to affect areas currently designated as biodiversity priority areas [63].

#### 2.5.3. Impact of CC on Pests, Diseases and Mycotoxigenic Fungi

In studies on pests and diseases, negative results of indirect climate-related impacts on coffee production were reported (34 papers). These included, from one side, the expected growth in the distribution and reproductive rates of pests such as the coffee berry borer [63] and coffee white stem borer [69,70]; from another side, increasing infestation of the coffee nematode (races of Meloidogyne incognita) and leaf miner (Leucoptera coffeella) in Brazil as a result of a higher monthly generation rate [53]. Numerous production regions in Colombia, Central America, and Nicaragua have been severely impacted by diseases such as coffee rust [58,71]. There were projected decreases in the incubation period of coffee rust, which could lead to more severe epidemics [72] and future pollinator richness in Latin America [62], which could have an impact on coffee production.

In contrast, one manuscript revealed a decrease in favourable areas for phoma leaf spot of coffee in Brazil over the next three decades (2020, 2050, and 2080) and a change in the temporal distribution of phoma leaf spot [73]. Another study indicated that the significance of coffee white stem borer (CWB) in one Zimbabwean district could decline by 2080 [69]. Bebber et al. 2016, [74] suggested that weather conditions have become less favourable for coffee leaf rust (CLR) in recent years, based on the decline in mean daily leaf wetness duration (LWD), which has resulted in a reduction in the daily CLR risk. Therefore, it is suggested that the decrease in canopy surface water may have helped end the epidemic.

Jaramillo et al. 2013, [75] indicated the impact on plant-insect interactions and agricultural yield, while Clough et al. 2017 [76] investigated the significance of ant-mediated interactions for pests and diseases incidence and agroforest productivity, showing that the role of ants as predators at intermediate altitudes might change as the temperature increases. Conversely, the increased pressure exerted by invasive ants threatens the crop. According to others [77], pollination and bee populations will change due to climate change’s direct and indirect effects on coffee yield. Fewer manuscripts (five papers) reported the effect of climate change on mycotoxigenic fungi, revealing that CC factors may stimulate mycotoxin production. Therefore, the likelihood of *Fusarium* and *Aspergillus* species producing mycotoxins will increase. Overall, the growth and production potential of Ochratoxin A (OTA) by *Aspergillus carbonarius* and *Aspergillus ochraceus*, as well as aflatoxins by *Aspergillus flavus*, which are more toxic than OTA, may become predominant, leading to an increase in food insecurity surrounding coffee production [78,79,80]. Therefore, Arabica and robusta will grow in less suitable climates, increasing plant stress and susceptibility to fungal infection and mycotoxin contamination [79].

#### 2.5.4. Impact of CC on the Physiological Response of Coffee Plants

In the present study, there was also research on the effects of various environmental changes, such as cold, high air temperatures, and drought, which caused alteration of the plants’ physiological performance (25 papers). Environmental limiting conditions stress coffee plants and have a global negative impact on biochemical reactions, as well as morphological, phenological, and other developmental characteristics by promoting cellular damage, disruption of ionic and osmotic homeostasis, oxidative stress, lipoperoxidation of membranes, protein degradation, etc., which could lead to lethal injuries to the stem, roots, and leaves. Moreover, additional adverse effects on coffee plants included a reduction in net photosynthesis, transpiration rate, net carbon assimilation rate, and an increase in long-term water-use efficiency (WUE). These effects may lead to crop yield losses and modifications in the quality and postharvest preservation of coffee products, as well as the risk of pest and disease attacks [81,82,83,84,85]. In addition, significant alterations may occur in the expression of genes associated with abiotic stress and senescence.

#### 2.5.5. Impact of CC on Coffee and Bean Quality and Farmers’ Socio-Economic Condition

Several studies have directly or indirectly highlighted the effects of climate change on coffee crops and bean quality (seven papers) and coffee producers (thirty articles). Climate variability causes declines in crop yield and alterations in the quality and preservation of coffee products [82]. Moreover, a study (conducted in Nicaragua) suggested that the quality of coffee beans may be adversely affected by increasing altitude; by 2050, the overall capacity to produce acidic and flavourful coffee beans will decline [21]. The study of Joët et al. 2010 [86] found that altitude positively affected the glucose content, while the sorbitol content after wet processing was directly proportional to the glucose content of fresh seeds. Therefore, the changes affecting the coffee plant and bean quality will impact the final beverage. However, several subcomponents of coffee bean defects (such as mouldy beans and insect damage) were also associated with the climate during the early and late growing seasons [87].

As documented in the literature, extreme weather events result in a less favourable climate for the production of high-quality coffee and a reduction in the producer’s income, as well as an increase in the financial and planning costs caused by a rise in the risk of this activity due to the significant uncertainty in climate scenarios [54,61]. Crop production and food and income security for farmers were limited by several socio-economic factors and climatic constraints [88]. As a result, economic losses, increased food insecurity, malnutrition, migration of farmers to other regions, and expansion of different land uses with less biodiversity value are likely to occur at the expense of forest cover [61,89]. The effects of these stresses on coffee production and livelihoods have already decreased exportable coffee production [90].

#### 2.5.6. The Mitigating Effect of [CO_2_]

This review comprised 29 articles discussing the impact of elevated [CO_2_] levels on the physiology and productivity of coffee agrosystems. According to the majority of these studies, some coffee-growing regions may benefit from increased [CO_2_], which may increase the photosynthetic rate [91] and heat tolerance of the plant, resulting in increased crop growth and yield [92,93]. [CO_2_] can significantly reduce heat stress on this crop by boosting plant vigour and resistance [94]. Under high [CO_2_], however, the latent period of coffee leaf rust and the incidence of leaf miners (*Leucoptera coffeella*) during periods of high infestation has decreased [72,95].

Simultaneously, [CO_2_] and irrigation conditions (pertaining to carbohydrates, chlorogenic acids, and caffeine) induce metabolic changes [96]. The CO_2_ factor had a greater influence on the metabolome of *Coffea arabica* beans than on water availability, resulting in elevated levels of quinic acid/chlorogenic acids, malic acid, and kahweol/cafestol [97]. The CO_2_-enriched plants were found to contain significantly higher concentrations of phenolic acids and caffeine-like compounds, indicating the successful metabolic adaptation of CO_2_-enriched Arabica coffee beans to future droughts [98]. In addition, lipid profile modifications in chloroplast membranes are expected to contribute to the coffee plant’s long-term acclimation to climate change under high [CO_2_] levels [99]. Others [100] reported that, besides the overall positive effect of [CO_2_] on mitigating the harmful CC impacts on the coffee crop, [CO_2_] reduced the concentration of chlorogenic acid (5-CQA) in *Coffea arabica* during the dry season when mites and other pests are prevalent. However, the diversity and abundance of mites in coffee leaves were not affected by [CO_2_], but mite diversity was strongly correlated with seasonal variation in coffee leaf phenolics. Consequently, it affects the quality of coffee beverages [100].

Elevated carbon concentration might enhance the photosynthetic process and increase yield [93,95], thereby mitigating, at least in part, the adverse effects of warming conditions on coffee yield [55]. However, Moat et al. 2017 [101] argued that increasing drought stress and the potential effects of deforestation on the local climate could eventually outweigh this positive effect. These interactions are context-dependent and thus require further investigation.

#### 2.5.7. Impact of CC on Ecosystem Services

Climate change will have either direct or indirect effects on the ecosystem. According to reports, significant shifts in areas suitable for coffee production within the next three decades may result in land conflicts between coffee production and nature conservation [102]. In addition, significant economic losses will occur throughout the coffee supply chain and the loss of ecosystem services [103]. Thus, the present review included fewer references (two papers) that specifically document the effect on ecosystems, mainly ecosystem services. In particular, minor literature work focused on the regulating services (disease regulating) or supporting services (biodiversity metrics, pollination and primary production).

Jaramillo et al. 2013, [75] linked the impact on ecosystems and ecosystem services, such as plant-insect interactions, to agricultural productivity as a consequence. Others [18] reviewed the literature on two crucial and interacting ecosystem services that regulate coffee production: bird control of a beetle infestation and bee pollination. Studies show that climate change and habitat loss will increase the extinctions of land bird species and decrease pest control, triggering the coffee berry borer (CBB) survival and distribution. Shifts in precipitation influence flowering phenology, affecting the diversity of visiting bee species and fruit sets. Therefore, anticipated changes in the spatial distributions of coffee and bees presume that coffee farms may experience pollinator deficits in the future [18]. Imbach et al 2017 [62] showed that the geographic range shift affecting the coffee lands would affect the pollinators in either a positive (10–22%) or a negative (34–51%) coupling depending on the area. The average number of bees will decline by 8–18% in future coffee-suitable regions. In 31–33% of prospective coffee distribution regions, bee abundance decreases, and coffee suitability rises.

However, the authors failed to present the results according to the impact types using the recognised ecosystem services framework [29].

Hence, the present review lacked studies that specifically document the relationship between the different ecosystem services (provisioning services, regulating services, supporting services and cultural services).

## 3. Discussion

Existing research is concentrated primarily on Arabica species since it can be more severely affected by CC, with reported declines, while robusta species may instead show some increase in productivity with some warming. However, findings show that each 1 °C increase above the mean minimum temperature of 16.2 °C during the growing season reduces robusta production by 350–460 kg ha^−1^, or 14% [42].

The majority of the reported effects of climate change on coffee production were negative. These included reducing suitable areas for coffee cultivation and production, farmers’ income and well-being, and increasing infestations and distribution of insect pests and diseases that reduce coffee berry quality and yield. However, there were also positive impacts, such as increased suitability for coffee production in new regions, especially at higher elevations and elevated CO_2_ concentrations, which mitigates the adverse effects of CC on the coffee crop.

A recent study included the vapor pressure deficit (VPD) impacts on global Arabica coffee productivity. It indicated that VPD during fruit development is a major indication of global coffee productivity, with the yield dropping fast above 0.82 kPa. Therefore, the incorporation and the determination of thresholds of VPD appear crucial for comprehending the effects of climate change on coffee and designing adaptation strategies [10].

Farmers, the coffee industry, and the global coffee supply are vulnerable to climate change. Socio-economic difficulties present further challenges, frequently preventing farmers from acquiring the resources and skills necessary to adopt resilient agricultural practices. Several qualitative studies have shown that while most farmers were aware of the effects of climate on their farming and livelihoods, they did not adopt these measures into their management practices [104,105]. However, the literature identifies various adaptation measures for managing climate-driven impacts on coffee production. Recent increases in Brazil’s crop production and yield have been linked to adopting new technologies [106]. According to some [107], warming may be less detrimental to the suitability of coffee than previously estimated, at least under the conditions of an adequate water supply.

Furthermore, several countries may face climate change risk that could permanently harm one of their economies’ essential aspects: coffee production. Some countries can take the necessary precautions to mitigate the effects of climate change, while others cannot. Thus, to help coffee smallholders adapt, it is crucial to combine suitable policy measures, technical solutions, research results, and best practices recommendations.

Essential for sustainable adaptation and resilience techniques and strategies for coffee crops, the literature failed to provide the results according to impact categories using the ecosystem services framework [29]. Accordingly, the literature has relied chiefly on provisioning services, such as production, while limited research focused on other ecosystem services such as regulating or supporting. To ensure the provision of vital ecosystem services, ecosystem functions must be supported and maintained, and biodiversity must be protected. Thus, more specified research on the interrelation of ecosystem services and biodiversity is required, following the ecosystem services framework proposed by the Millenium Ecosystem Assessment [29]. This should investigate the impacts of climate change and assess the expected risk by considering the complex interactions between climate, ecosystems, and human society. Current research highlights changes in coffee-growing areas’ distribution and pays less attention to coffee yield and pest and disease distribution. Due to the possibility that some of the significant coffee pests and diseases will benefit from rising temperatures, more research is required on their responses to changing climatic conditions and adaptation mechanisms to minimise the coffee crop’s exposure and vulnerability to these risks. More research is needed on the direct and indirect effects of climate change on coffee yield, especially in Asia and on robusta coffee, and on the efficacy of adaption in maintaining the industry’s sustainability and viability. More knowledge about positive influences on coffee production is needed, such as the long-term potential for elevated atmospheric carbon concentration to offset warmer conditions and pollination activities. In addition, a better understanding of the future distribution of coffee-favourable space, considering potential ecological and socio-economic impacts and associated opportunities and challenges, is recommended to support sustainable coffee development better.

## 4. Materials and Methods

The CEE guidelines [23] describe the systematic review convention, which breaks down the primary research question into definable components known as PICO or PECO. The PICO elements also determine the keywords to formulate the search terms as the methodology requires. The review team has agreed on the PICO elements defined in Table 2. Once developed, the team trialed 16/06/21 different search terms (Table 3) using the three major research engines (Web of Science, Scopus, and Science Direct). The review team avoided the excessive use of search operators such as wildcards, booleans, braces, etc., to avoid incompatibilities between different engines.

Besides the three academic database sources, the systematic review searched a list of websites and organisation websites that the team considered relevant to the study (Table 4). A maximum of 50 ‘hits’ was considered for the full review from each search website.

The review team screened all literature retrieved using the study inclusion criteria as follows: (i) relevant subjects (any countries/regions; any scale, from field to regional; any coffee agrosystems including small-scale and commercial systems), (ii) type of intervention (climate change emission scenarios for time slices up to 2100; emission scenarios based on IPCC models; projected changes in mean, total or seasonality), (iii) comparator (compares future outcomes with baseline outcomes), (iv) method (qualitative research, surveys, controlled experiments, biophysical modelling, etc.), (v) outcomes (records that consider the change in crop suitability, performance, variability, sustainability).

The potential “effect modifiers” were inevitable given the limited primary data available and the variability in study contexts modelling tools and impacts (e.g., different emission scenarios, crop species/varieties, production practices and techniques, agroecological conditions, etc.). Therefore, subject to this caveat, the team avoided the meta-analysis and opted for a narrative synthesis of results with some quantitative evidence. A narrative approach suits references with broad subject content and a disparate range of potential outcomes. It also can highlight, for stakeholders and decision-makers, existing knowledge gaps in the subject and areas suitable for targeting [24,25,30]. The review team exercised high care in interpreting records reporting mitigation and adaptation to climate change impacts across coffee agrosystems to avoid any source of bias.

The data extraction took place on 21 July 2021, without limiting the database search to a specific timespan to explore all the literature available in the field. The approach extracted all relevant data based on the ‘outcome’ search terms and exported them into “Mendeley” (a bibliographic software package) before assessing relevance using inclusion criteria.

We only considered literature published in English for filtering. We tabulated information using spreadsheets (MS Excel) during the full-text review. The data extraction process was carefully documented for transparency, reporting any reasons for data heterogeneity.

## 5. Conclusions

This review considers information from the available literature concerning the effects of climate change and variability on coffee production. This paper includes documents that cover countries from three continents (America, Africa, and Asia). The number of records available from each continent differs, with most of the studies being in America.

Studies have already demonstrated that coffee cultivation will face declines in coffee yield, shifts in areas of suitability, and an increase in the distribution of pests and diseases. Nevertheless, even though this review assessed a relatively large number of studies, there was no clear evidence on how climate change affects coffee production’s ecosystem services. The current literature evaluates and analyses various climate change impacts on single services using qualitative and quantitative methodologies. In contrast, research lacked documentation on the precise relationship between the potential risks of CC and coffee ecosystem services.

Consequently, further work is needed on ecosystem services, both direct (provisioning and cultural) and indirect (supporting and regulating), and their interrelation, to assess the impacts of climate change on coffee agro-systems following the ecosystem services framework.

## 6. Future Research Recommendation

Given the importance of ecosystem services for the achievement of sustainable development and climate-resilience goals, their assessment is pivotal to inform short- and medium-term planning decisions, in particular for commodities such as coffee of strategic social and economic relevance.

Therefore, the main recommendation of this study is to fill the research gap highlighted by this systematic review, by promoting the scientific research toward assessing the multiple impacts on the ecosystem services provided by coffee cultivation by analyzing their interrelationships as provided by the ecosystem services framework.

In addition, it is essential to assess how the provision of these services may change under future climate change conditions.

Moreover, stakeholders should be made aware of the importance of assessing coffee-related ecosystem services in order to target investments and prioritize actions to increase the sustainability and resilience of coffee systems worldwide.

## Figures and Tables

**Table 1:** Top-producing countries in the world in 2019.

Rank	Country	Area Harvested (ha)	Production (Tonnes)
1	Brazil	1,823,403	3,009,402
2	Indonesia	1,258,032	760,963
3	Côte d’Ivoire	953,972	67,697
4	Colombia	853,700	885,120
5	Ethiopia	758,523	482,561
6	Mexico	629,300	165,712
7	Vietnam	622,637	1,683,971
8	Uganda	469,364	254,088
9	Honduras	420,957	476,345
10	India	416,741	319,500
11	Peru	359,508	363,291
12	Guatemala	308,217	225,000

Source: [7].

**Table 2:** The breakdown of the research question into PICO components and related keywords.

PICO	Description	Keywords
Population	Coffee production, focusing on agrosystems and bean production but excluding the processing phases following the postharvest The review will include all agrosystems that could be found in the areas included in the study The review will consider no time scale It will include all scenarios investigated in the literature The study will not be limited to a specific geographical area and will include all continents and countries known for coffee production, from Asia to Africa to America.	Coffee, crop, tree, production, agrosystem, farm
Intervention	Coffee agrosystems have generated social, economic and environmental benefits and impacts in rural areas where they are established Climate change, as projected by IPCC and various GCMs available in the literature could have negative and/or positive impacts on coffee agrosystems and trade-offs between different ecosystem services Climate variables to be included are temperature (mean, seasonal variation), rainfall (mean annual and seasonality), and changes in CO_2_ concentration	Climate change, temperature, rainfall, CO_2_
Comparator	Baseline climate, typically 1961-90 (note: there will be other defined ‘baselines’ reported in the literature which may constitute an ‘effect modifier’)	Baseline, scenarios
Outcome	Provisioning services: average yields and yield variability, yield quality, new varieties Regulating services: irrigation water needs, water pollution, carbon emission and/or sequestration; land suitability Supporting services: soil quality, nutrients’ cycle, agrosystems’ biodiversity Cultural services: historical and traditional value of land-use change	Provisioning services, regulating services, supporting services, cultural services, yield, fertiliser, nutrition, irrigation crop failure, disease, varieties, drought, soil degradation, biodiversity, salinity, land suitability, poverty alleviation, farm income

**Table 3:** Development, trial, refinement, and screening of search terms. The number of papers reached per search term are included. The search term stressed in bold represents the ideal choice.

Search Term	Science Direct	WoS (All Fields)	Scopus (Title-Abs-Key)	Comments
“climate change” AND coffee	5756	561	564	The search term is too broad. It might include the adaptation and resilience of coffee to climate change
coffee AND ecosystem	8794	757	883	Too broad. It also excludes climate change and its impacts from the results.
“climate change” AND coffee AND impact	4961	274	237	A good search term. A reasonable number of hits includes all the words needed to answer the research question.
**climate AND coffee AND impact**	**11,830**	**360**	**282**	**A good search term. A reasonable number of hits includes all the words needed to answer the research question.**
climate AND coffee AND impact AND ecosystem	4123	79	46	A good search term but including ecosystem in the keyword would exclude all research on climate change impacts on coffee that did not specifically address ecosystem services.
climate AND coffee AND (temperature OR rainfall OR CO_2_)	10,715	324	340	The search term only relates climate change to coffee without considering impacts.
climate AND coffee AND (ecosystem OR nutrition OR irrigation OR failure OR disease OR drought OR soil OR salinity OR biodiversity OR variety OR income OR poverty)	–	580	576	Too many boolean connectors. ScienceDirect did not search it. Web of Science and Scopus found a reasonable number of hits.
climate AND (temperature OR rainfall OR CO_2_) AND coffee AND (crop OR tree OR production OR farm OR agrosystem) AND impact AND (ecosystem OR nutrition OR irrigation OR failure OR disease OR drought OR soil OR salinity OR biodiversity OR variety OR income OR poverty)	–	86	72	Too many boolean connectors. Science Direct was not able to perform such a search. In Web of Science and Scopus this keyword was too restrictive and retrieved a limited number of documents for a systematic review.
coffee AND (ecosystem OR nutrition OR irrigation OR failure OR disease OR drought OR soil OR salinity OR biodiversity OR variety OR income OR poverty)	–	12,814	13,975	Too many boolean connectors. ScienceDirect was not able to perform such a search. Web of Science and Scopus retrieved too many documents, too broad for a systematic review and may include coffee trade and industry and varietal improvement without any correlation to climate change and its impacts.

**Table 4:** List of academic database sources and websites used.

Database Sources	Search Websites	Organisation Websites
Web of Science (WoS) Scopus ScienceDirect	google.com googlescholar.com	World Bank FAO Consultative Group on International Agricultural Research (CGIAR) The International Center for Tropical Agriculture (CIAT) International Coffee Organisation Coffee Research Institute Global Coffee Platform International Fund for Agricultural Development (IFAD) Natural Resources Institute Climate Institute Coffee & Climate International Trade Centre (ITC) FairTrade International

## Data Availability

The data presented in this study are available on request from the corresponding author.
